# International Consortium on Ageing-Related Pathologies (ICCARP) Audiovestibular Group: fostering international consensus to refine International Classification of Diseases (ICD-11) codes for hearing loss across the life course

**DOI:** 10.1007/s11357-025-01742-2

**Published:** 2025-06-28

**Authors:** Dialechti Tsimpida, Michael A. Akeroyd, Barry L. Bentley, Shuvarthi Bhattacharjee, Michael R. Bowl, Emma Broome, Stuart R. G. Calimport, Sian Calvert, Gary Christopher, Tom Dening, Silvia Di Bonaventura, Ankita Goswami, Spyridon Gougousis, Paul J. Govaerts, Mini Gupta, Helen Henshaw, Robert T. R. Huckstepp, Vasiliki Maria Iliadou, Theano K. Koutsimani, Morag A. Lewis, Frank R. Lin, Cecilia Luisa Miotto, Lisa S. Nolan, Helen E. Nuttall, Chukwuebuka Prince Onyekere, Mukovhe Phanguphangu, Christopher J. Plack, Ramasamy S. Raghavan, Nicholas S. Reed, Konstantina Rova, Karen P. Steel, Robert J. Stokroos, De Wet Swanepoel, Agnieszka J. Szczepek, Susan L. Whitney

**Affiliations:** 1 Centre for Research On Ageing, University of Southampton, Southampton, UK; 2 Department of Gerontology, University of Southampton, Southampton, UK; 3 National Institute for Health and Care Research (NIHR) Nottingham Biomedical Research Centre, Nottingham, UK; 4 Hearing Sciences, Mental Health and Clinical Neurosciences, School of Medicine, Faculty of Medicine and Health Sciences, University of Nottingham, Nottingham, UK; 5 Cardiff Metropolitan University, Cardiff, UK; 6 Collaboration for the Advancement of Sustainable Medical Innovation (CASMI), University College London, London, UK; 7 Center for Engineering in Medicine and Surgery, Harvard Medical School, Boston, MA USA; 8 Department of Surgery, Massachusetts General Hospital, Harvard Medical School, Boston, MA USA; 9 Shriners Children’s, Boston, MA USA; 10 School of Education, Sport and Health Sciences, University of Brighton, Brighton, UK; 11 UCL Ear Institute, University College London, London, UK; 12 Centre for Ageing and Dementia Research, Swansea University, Wales, UK; 13 Mental Health and Clinical Neurosciences, School of Medicine, Faculty of Medicine and Health Sciences, University of Nottingham, Nottingham, UK; 14 University College London Hospitals NHS Foundation Trust, London, UK; 15 Norwich Medical School, University of East Anglia, Norwich, UK; 16 Ear Nose Throat Department, General Hospital of Thessaloniki George Papanikolaou, Thessaloniki, Greece; 17 The Eargroup, Antwerp, Belgium; 18 Department of Translational Neurosciences, University of Antwerp Faculty of Medicine and Health Science, Antwerp, Belgium; 19 Language and Hearing Center Amsterdam, Free University Amsterdam, Amsterdam, The Netherlands; 20 All Ears Hearing & Tinnitus Clinic, Audiology Australia, Mount Waverley, Victoria, Australia; 21 School of Life Sciences, University of Warwick, Coventry, UK; 22 Medical School of Aristotle University, Thessaloniki, Greece; 23 Wolfson Sensory, Pain, and Regeneration Centre, King’s College London, London, UK; 24 Department of Otolaryngology–Head and Neck Surgery, Johns Hopkins School of Medicine, Baltimore, MD USA; 25 Department of Psychology, Lancaster University, Bailrigg, UK; 26 School of Kinesiology and Health Science, Faculty of Health, York University, Toronto, Canada; 27 Department of Audiology, College of Health Sciences, University of KwaZulu-Natal, Durban, South Africa; 28 Department of Family Medicine, College of Health Sciences, University of KwaZulu-Natal, Durban, South Africa; 29 Manchester Centre for Audiology and Deafness, The University of Manchester, Manchester, UK; 30 Royal Surrey Hospitals NHS Foundation Trust, Guildford, Surrey UK; 31 Departments of Otolaryngology-Head and Neck Surgery and Population Health, New York University Grossman School of Medicine, New York, NY USA; 32 University Medical Center Utrecht, Utrecht, The Netherlands; 33 Department of Speech-Language Pathology and Audiology, University of Pretoria, Pretoria, South Africa; 34 Department of Otolaryngology, Head and Neck Surgery, Charité Universitätsmedizin Berlin, Berlin, Germany; 35 School of Health and Rehabilitation Sciences, University of Pittsburgh, Pittsburgh, USA

Dear Editors,

Following the World Health Organization’s (WHO’s) decision to classify age-related aetiologies [1], and a global call for action to systematically classify the pathologies of ageing [2], the International Consortium to Classify Ageing-Related Pathologies (ICCARP) was established in 2023 under the leadership of Cardiff Metropolitan University [3, 4]. Within this consortium, the Audiovestibular Group is actively working to refine the classification of hearing and balance disorders, aligning with the WHO’s commitment to enhance diagnostic frameworks. This effort coincided with the release of the 2025 edition of the International Classification of Diseases 11th Revision (ICD-11) on 14th February 2025 [5].

 The updated ICD-11 provides a globally standardised system for diagnosing, reporting, and monitoring diseases, injuries, and causes of death, guiding clinical decision-making, research, and public health policy worldwide. Notably, hearing disorders are now categorised under “Disorders with Hearing Impairment” (AB50-AB5Z), with detailed subcategories for specific conditions, procedures, and functional assessments, as illustrated in Tables 1 and 2 [6].

**Table 1:** Codes for disorders with hearing impairment in the international classification of diseases for mortality and morbidity statistics, 11th revision

ICD-11 code	Description
AB50	Congenital hearing impairment
AB50.0	Congenital conductive hearing loss
AB50.1	Congenital sensorineural hearing loss
AB50.2	Congenital mixed conductive and sensorineural hearing loss
AB50.Y	Other specified congenital hearing impairment
AB50.Z	Congenital hearing impairment, unspecified
AB51	Acquired hearing impairment
AB51.0	Acquired conductive hearing loss
AB51.1	Acquired sensorineural hearing loss
AB51.2	Acquired mixed conductive and sensorineural hearing loss
AB51.Y	Other specified acquired hearing impairment
AB51.Z	Acquired hearing impairment, unspecified
AB52	Deafness not otherwise specified
AB53	Ototoxic hearing loss
AB54	Presbycusis
AB55	Sudden idiopathic hearing loss
AB56	Hereditary hearing loss
AB57	Auditory synaptopathy or neuropathy
AB5Y	Other specified disorders with hearing impairment
AB5Z	Disorders with hearing impairment, unspecified

**Table 2:** Related codes for specific hearing conditions, procedures, and functional assessments in the international classification of diseases for mortality and morbidity statistics, 11th revision

ICD-11 code	Description
LA22	Structural developmental anomalies of ear causing hearing impairment
LA22.Y	Other specified structural developmental anomalies of ear causing hearing impairment
LA22.Z	Structural developmental anomalies of ear causing hearing impairment, unspecified
LD2H.1	Neuropathy with hearing impairment
QA00.7	Examination of ears and hearing
QB30.0	Adjustment or management of implanted hearing device
QB30.0Y	Adjustment or management of other implanted hearing device
QB30.0Z	Adjustment or management of implanted hearing device, unspecified
QB31.4	Fitting or adjustment of hearing aid
QB51.B	Presence of external hearing-aid
VE01	Hearing and vestibular functions [BMDS]
VV11	Hearing and vestibular functions

In the current edition, the terms *hearing loss* and *hearing impairment* are used interchangeably, reflecting common practice in both clinical and academic settings. Distinction between hearing loss and hearing impairment should be made to ensure appropriate use of hearing loss as a part of hearing impairment. While *hearing loss* often refers specifically to audiometric threshold shifts, *hearing impairment* serves as a broader term encompassing any difficulty in hearing, including cases not well predicted by audiograms (e.g. poor speech-in-noise performance, auditory neuropathy spectrum disorder, auditory processing disorder) [7–9].

## Changes from ICD-10 to ICD-11 classification for hearing loss

The previous ICD-10 system classified hearing loss into two main categories: type-based (H90) and cause-based (H91) [10]. This distinction allowed clinicians to differentiate between the type of hearing loss (e.g. conductive, sensorineural, mixed) and its underlying cause (e.g. ototoxicity, sudden idiopathic hearing loss).

The updated ICD-11 system reorganises the classification under “Disorders with Hearing Impairment” (AB50-AB5Z), introducing distinct codes for congenital (AB50) and acquired (AB51) hearing impairment [6]. While this refined approach supports clinical practice and research, a challenge arises with the separate classification of presbycusis (AB54) alongside the two broader categories of congenital (AB50) and acquired hearing impairment (AB51; see Table 1).

## The challenge with code AB54 (presbycusis) and redundancy in the system

ICD-11 assigns presbycusis a separate code (AB54), categorising it as “sensorineural hearing impairment in elderly individuals,” distinct from other types of acquired hearing loss (AB51). This separation creates inconsistency, as it overlooks the broader spectrum of sensorineural hearing impairments that can occur across all age groups [11] and reinforces the widespread misconception that hearing loss is an inevitable consequence of chronological ageing. Research increasingly shows that what is often termed as “presbycusis” is often the result of multiple factors beyond age, including noise exposure [12], ototoxic medications [13], and underlying conditions such as diabetes [14] or cardiovascular disease [15]. Furthermore, hearing outcomes in later life vary significantly, influenced by factors such as socioeconomic position and geographical location [16, 17].

The inclusion of AB54 introduces redundancy within the classification system, since the existing category for acquired hearing loss (AB51.0–2) already accommodates age-related factors [18], therefore making this separate classification unnecessary. Importantly, studies indicate that only 10–20% of the variance in acquired hearing loss can be attributed solely to chronological age [17, 19], suggesting that other factors across the lifespan play a significant role in what is commonly classified as “presbycusis”. Emerging evidence points to the idea that variations in hearing decline are more closely linked to differential and cumulative exposure to harmful environmental and other risk factors throughout an individual’s life course [16, 17, 20, 21].

## Clinical and public health concerns

The classification of presbycusis as a standalone category (AB54) has significant clinical and public health implications. While some physiological changes occur with age, ageing is a natural process, not a disease. Age-based approaches risk oversimplifying hearing loss, often medicalising variations in sensory function that occur as part of the human lifespan and underestimating its multifactorial nature. This overemphasis can obscure modifiable risk factors and hinder understanding of prevention, and the development of more comprehensive audiological interventions and healthcare planning [22].

By designating hearing loss in older adults as a separate entity—under the label of presbycusis—the system risks oversimplifying diagnoses and diminishing clinical attentiveness. This classification can inadvertently reduce the focus on prevention and obscure modifiable risk factors, thereby undermining public health initiatives aimed at mitigating hearing loss in ageing populations. Such an approach contrasts with the World Health Organization’s emphasis on using routine health information systems to support evidence-based decision-making in health policy, vmanagement, and clinical care [23].

Rather than investigating underlying or contributing factors—such as noise exposure, ototoxic medications, or even genetic predispositions—clinicians may default to presbycusis as the explanation for hearing loss in older adults. Crucially, this tendency not only greatly compromises diagnostic accuracy but also increases the likelihood of missing treatable conditions. For example, hearing loss in ageing populations could be caused by significant underlying conditions such as neuromas, as well as neurological disorders like amyotrophic lateral sclerosis (ALS) [24] and multiple sclerosis (MS) [25]. These conditions require more in-depth investigations and targeted interventions, such as the surgical removal of neuromas, which hearing aids or cochlear implants would not effectively manage. Failing to address these conditions in a timely manner could delay appropriate treatment, leading to dire consequences for patients and further escalating healthcare costs associated with the management of advanced pathologies [26].

Moreover, framing hearing loss in later life as inevitable reinforces harmful stereotypes and undermines preventative care, thereby discouraging further investigation into modifiable causes, reducing help-seeking behaviour, and delaying diagnosis and treatment [27]. Conditions such as noise-induced hearing loss, autoimmune-related auditory dysfunction, and medication-induced ototoxicity require specific management strategies [28], yet they may be overlooked if hearing loss is automatically attributed to presbycusis.

In addition, the standalone categorisation of presbycusis limits inference about the severity of the condition. While progress has been made in classifying the type of hearing loss, information on the severity could guide public health and clinical interventions, such as guiding policy for coverage for interventions that meet the needs of different degrees of hearing loss. Moreover, severity information could reshape the way individuals view hearing loss from a broad, binary event to align with the reality that hearing loss changes across the lifespan and encourage individual action on the prevention of further loss.

Finally, redundancy within the classification system introduces ambiguity, increasing the risk of inconsistent coding practices. This variability can affect the accuracy of diagnoses, reporting, and data collection, which in turn hinders epidemiological research essential for understanding the prevalence and risk factors of hearing loss in older populations, ultimately impacting broader public health efforts. Inconsistent classification may obscure important epidemiological data and hinder research into the various risk factors for hearing loss in older populations. This misclassification can disrupt surveillance, policy development, and preventative strategies, ultimately reducing the effectiveness of public health strategies aimed at both preventing and effectively managing hearing loss across the life course. Such an approach undermines efforts to normalise hearing loss diagnosis and management across all age groups, which risks creating age-based disparities [29].

## Proposed solution: elimination of AB54

Based on the above considerations, the ICCARP Audiovestibular Group *recommends the elimination of AB54 (Classification of presbycusis as a standalone category)* *and the use of the existing AB51 category to classify acquired hearing loss regardless of the patient’s age, incorporating an extension code to specify severity levels that align with the current WHO categories (i.e., mild, moderate, moderately severe, severe, profound)* [30]***.*** This change would reduce redundancy and enable more accurate diagnostic practices, shifting the focus away from assumptions based on chronological age. It would support collection of epidemiological data and research into specific causes of hearing loss, leading to targeted prevention strategies. This revision would maintain the logical structure of Table 1, allow for more precise classification using existing codes, and better reflect the multifactorial nature of hearing loss in older adults.

For example, an older adult experiencing progressive hearing loss could be classified under AB51.1 (Acquired Sensorineural Hearing Loss) with an extension code to specify severity and additional codes from Table 2 when appropriate (such as QB31.4 for hearing aid fitting or LD2H.1 for cases involving neuropathy). This classification would provide a more accurate framework for diagnosis and treatment.

Addressing this issue within the classification system would not only improve diagnostic accuracy but also support a more individualised approach to treatment. By recognising the multifactorial nature of hearing loss in ageing populations, ICD-11 could facilitate better clinical decision-making, enhance public health strategies, and ensure that preventable or treatable causes of hearing loss are not overlooked, regardless of a person's age.

## Alignment with WHO objectives

This proposed revision aligns with WHO’s commitment to evidence-based practice and its mission to provide accurate health information. It would support both WHO’s Global Health Strategy and Fourteenth General Programme of Work 2025–2028 and progress towards relevant Sustainable Development Goals by improving our understanding, prevention, and treatment of hearing loss across all age groups [31].

This change would harmonise with the existing structure of ICD-11 [6], where Table 2 already provides complementary codes for specific conditions, procedures, and functional assessments. The removal of AB54 would not create gaps in classification but would instead encourage more precise use of the remaining codes. Finally, this revision would also align with WHO’s broader goals for hearing health, including the *World Report on Hearing* [30], which advocates for a shift towards evidence-based strategies for addressing hearing loss worldwide.

## Conclusion

The transition from an age-based to an onset- and aetiology-based classification system would represent a significant advancement in how we understand, diagnose, and treat hearing loss in older adults. By eliminating AB54 and using the existing AB51 category for all acquired hearing loss, we would more accurately reflect the multifactorial nature of hearing loss—including the role of tissue and organ senescence across all age groups—enhance clinical practice, and support public health strategies.

By adopting an onset- and aetiology-based approach, we can not only improve diagnostic accuracy but also promote more person-centred models of hearing care that prioritise individuals’ communication needs and support their social inclusion.

This shift would lead to more precise diagnoses, improved data for research and public health planning, and ultimately, better health outcomes for individuals affected by hearing loss, regardless of age. We urge the WHO to consider this revision in the next updates of ICD-11, ensuring the system reflects the full complexity and diversity of hearing loss across the lifespan.

## Data Availability

The data utilised in this study are publicly available through the World Health Organization. They can be accessed via the *International Classification of Diseases, 11th Revision (ICD-11)* at the following link: https://icd.who.int/en.
